# Multivariate signals of population collapse in a high‐throughput ecological experiment

**DOI:** 10.1002/ecy.70197

**Published:** 2025-09-15

**Authors:** Francesco Cerini, John Jackson, Duncan O'Brien, Dylan Z. Childs, Christopher F. Clements

**Affiliations:** ^1^ Dipartimento Scienze Ecologiche e Biologiche Università della Tuscia Viterbo Italy; ^2^ School of Biological Sciences University of Bristol Bristol UK; ^3^ School of Biosciences University of Sheffield Sheffield South Yorkshire UK; ^4^ Department of Conservation Biology and Global Change Estación Biológica de Doñana (EBD‐CSIC) Seville Andalusia Spain

**Keywords:** behavior, conservation, early warning signals, experiment, extinction, morphology, multidimensional monitoring, *Paramecium*, population collapse

## Abstract

Anticipating population declines is a crucial goal of conservation ecology. Recent conceptual work suggests that populations facing growing stressors should exhibit sequential shifts in behavior, morphology, and abundance before declining to extinction. However, the lack of high‐resolution, multidimensional data has hindered empirical validation of this conceptual work. Using an autonomously monitored, high‐throughput experimental system, we generated individual‐based data on populations of the ciliate *Paramecium caudatum* forced to collapse due to increasingly stressful conditions. The gradual introduction of a pollutant elicited the predictable sequence of responses—declines in movement speed, followed by declines in body length, emergence of early warning signals of collapse, and finally, abundance declines. Conversely, a press disturbance generated by the introduction of predators did not induce this sequence. The time between the first detectable trait changes and population collapse depended on the statistical approach used, but the sequence remained consistent. Using general additive models, detectable behavioral signals in the polluted populations occurred one generation before abundance‐based early warning signals were detectable, and two generations before abundance decline. We highlight that multivariate monitoring, particularly individual‐based metrics, is crucial for forecasting population declines.

## INTRODUCTION

Human activities are the root cause of the drivers of biodiversity change, such as habitat loss and degradation, climatic change, overexploitation, and the introduction of pollutants and invasive species (Bonebrake et al., 2019; IPBES, 2019). These drivers, hereafter referred to as “stressors,” exert pressure on natural systems, contributing to the ongoing “sixth mass extinction” (Ceballos et al., 2015; Cowie et al., 2022) and increasing the risk of future abrupt ecosystem change (Botta et al., 2019; Pigot et al., 2023). Such abrupt change arises when species can no longer adapt or avoid stressors, resulting in fast declines in population sizes and ultimately in local extinctions, which can destabilize ecological networks and hamper ecosystem service provision (Brook et al., 2008; Strona, 2022). For example, the introduction of alien species or changes in land use may cause population decline within one or two decades for reptiles (Emery et al., 2021; Guiller et al., 2022), while climate anomalies can cause a substantial population reduction over a single season in butterflies (van Bergen et al., 2020). Similarly, water pollution or overfishing can induce near extinctions in fish populations, resulting in altered trophic networks and ecosystem instability (Demertzioglou et al., 2022; Houk & Musburger, 2013; Kidd et al., 2007). Consequently, our ability to reliably predict whether a given population is at risk of collapse remains a fundamental goal of biodiversity conservation. Here, we define collapse as a rapid and sustained decline from a pre‐existing population abundance state (e.g., equilibrium state or carrying capacity; Cerini, Childs, & Clements, 2023; Cumming & Peterson, 2017). The magnitude of such a decline may vary by context, but in ecology and conservation biology population declines of 80%–90% or more, relative to a reference state, are commonly used as operational thresholds for defining collapse (Aagaard et al., 2016; Cumming & Peterson, 2017; Keith et al., 2013).

Extensive efforts have been made to analyze and forecast population dynamics using the abundance of a population, either via empirically derived population models of extinction risk (e.g., population viability analysis; Chaudhary & Oli, 2020; Coulson et al., 2001; Jackson et al., 2019) or forecasting the occurrence of a smooth transcritical bifurcation using early warning signals (EWSs; Clements & Ozgul, 2018; Drake & Griffen, 2010). However, such tools are highly variable in their reliability (Brook et al., 2000; Butitta et al., 2017; Patterson et al., 2021; Su et al., 2021), particularly when data quality is poor (e.g., short time series not capturing demographic processes; Coulson et al., 2001) and model assumptions are not met (Boettiger & Hastings, 2012).

Most importantly, current tools are often limited in their forecast horizon: the upper limit of time in the future for effectively estimating ecological change (Clements & Ozgul, 2016a; Petchey et al., 2015). The definition of forecast horizon length can depend on the generation time of the organisms being studied. For example, detecting signals of collapse two generations before a decline in a short‐lived organism's abundance could be considered a long forecast horizon relative to that organism but not necessarily from a conservation perspective. Conversely, long‐lived organisms act at timescales in line with conservation operations, yet monitoring programmes often only observe part of such organisms' lifespan. In these terms, “short” forecast horizons hamper our ability to implement management actions and reverse population declines; therefore, increasing the time gap between the first observation of warning signals and the occurrence of collapse is a major goal in biodiversity monitoring (IPBES, 2019).

Recent advances have highlighted the value of incorporating data beyond that typically used in predictive ecology (abundance and demography), particularly individual‐based data (behavior and morphology), which are predicted to change rapidly in response to stressors (Cerini, Childs, & Clements, 2023; Clements & Ozgul, 2018; Keen et al., 2021; Sih, 2013). For example, including body size data in EWS frameworks increases the accuracy of signals inferring collapse and decreases the length of time series required to predict transcritical transitions (Clements et al., 2017; Clements & Ozgul, 2016a). Indeed, scaling from individual‐level processes to the population, from a basis of physiological responses to the environment (Brown et al., 2004; Fayet et al., 2021; Wikelski & Cooke, 2006), can provide a more complete picture of population change (Cerini, Childs, & Clements, 2023; Lewis et al., 2006; Pirotta et al., 2018). For example, environmental stressors influence physiological pathways, which in the short term can impact behavior; but over longer timescales, chronic stressors influence morphological traits and, ultimately, demographic rates and population dynamics (Guindre‐Parker & Rubenstein, 2021; Ozgul et al., 2010). Thus, downstream effects of stressor‐induced physiological changes acting on behavior and morphology create the opportunity to observe a sequence of signals before changes in population abundance occur, which we term the timeline to population collapse (Cerini, Childs, & Clements, 2023) or simply timeline to collapse. The timeline to collapse, integrating individual and population responses to stressors, lays the conceptual groundwork for an ecological monitoring framework applicable to species that are, or are suspected to be, under growing levels of stressors, with potential to improve the reliability of EWSs and generally the management of at‐risk populations.

The implementation of the timeline to collapse framework requires continuous monitoring of populations to build time series of multidimensional data from the individual to the population level (e.g., behavior, morphology, and abundance; Cerini, Childs, & Clements, 2023). Promisingly, the rise of autonomous monitoring in ecology has the potential to fill this multidimensional data gap (Besson et al., 2022; Cavender‐Bares et al., 2022), but despite this, an appropriate dataset to empirically test the timeline has previously not existed. Performing ecological experiments to characterize the occurrence of the timeline in real‐life systems is thus necessary to test the conceptual work.

Here, we employ a cutting‐edge autonomous monitoring system to collect multidimensional data on experimental protist populations that were driven to collapse by increasingly stressful environmental conditions. Specifically, we define collapse as the point at which abundance falls below 10% of a reference abundance after acclimatization. Our goal was to experimentally validate the occurrence of the timeline of signals before collapse and compare the timing of occurrence of individual‐level signals with classic EWSs. The conceptual framework assumes that a gradual increase from low to high stress levels (i.e., ramp disturbance; Lake, 2003) is the ideal condition to observe the timeline of changes, whereby individuals have time to implement phenotypic responses (Cerini, Childs, & Clements, 2023). In contrast, an immediate transition from unstressed to stressed conditions, characterized by a sudden, high‐magnitude stressor event (i.e., a press disturbance; Bender et al., 1984), might not generate an observable timeline to collapse. Thus, to test both scenarios, we induced a decline in populations of the widely used ciliate model *Paramecium caudatum* by means of: (1) gradually increasing the concentration of a pollutant (ramp disturbance), and (2) the introduction of a predatory species (press disturbance), while concurrently monitoring movement speed, body size, and abundance eight times every generation. We explored the statistical support for the timeline using two approaches, general additive models and piecewise regression models, which measured the temporal occurrence of changes in behavior, morphology, and abundance trends. We also explored support for widely used abundance EWSs. Our main predictions were that (1) using trait data would expand the time from when responses in control and treatment populations start diverging until the treatment populations collapse, and (2) responses would diverge in the order of behavior, morphology, EWSs, and finally abundance.

## MATERIALS AND METHODS

### Experimental protocol

A stock of *P. caudatum*, kept at Bristol University and originally purchased from Sciento (Manchester, UK), was raised in a temperature‐controlled room for 2 weeks at 18°C and 80% humidity in constant light. The growth medium consisted of crushed protozoa pellets (Blades Biological LTD) dissolved in Chalkley's solution at a concentration of 0.3 g L^−1^. We then autoclaved (Clements et al., 2013) and filtered the medium through Whatman no. 1 filter paper to improve media clarity and autoclaved it again. We inoculated this medium with two species of bacteria, *Bacillus subtilis* and *Pseudomonas fluorescens*. At these conditions, *P. caudatum* had a generation time of ~23 h.

We performed the experiment in microcosms consisting of rectangular patches (L5.6 × W3.6 × H1.6 cm), custom designed using FreeCAD 3D‐design software (https://www.freecad.org/) and 3D‐printed in clear PLA filament (Lulzbot TAZ 6). We painted the base of each microcosm with black acrylic paint and coated it in transparent epoxy resin to smooth the patch surface and prevent potential paint leaching. Each microcosm was filled with 6 mL of the medium described above, which was inoculated 48 h before the start of the experiment (day −2). On day 0, we added ~60 *P. caudatum* individuals to each microcosm and left them to grow for 14 days to reach a stable population size. Every day, we topped up the microcosms with autoclaved distilled water to replace evaporation. At the start of the third week, we started the stressor treatments. We had 10 replicates for each treatment, for a total of 30 microcosms. We used three treatments: a control treatment, a pollution treatment, and a predator treatment. In the control treatment, no stressor was applied to the populations. In the pollution treatment, an increasing quantity of a copper sulfate solution was added every day over a 10‐day period. Copper ions are toxic for aquatic ciliates (Madoni & Romeo, 2006) and have been previously used for experimental extinction tests (Sommer et al., 2017). The initial pollution quantity was calculated to reach 5% of a pretested copper concentration (0.6 mg L^−1^) in the experimental microcosm, lethal to a dense population of *P. caudatum*. Every day, we increased the copper concentration by adding another 1.5 μL (the initial quantity) of the copper sulfate solution to the previous day's one (i.e., a linear increase with a rate of 1.5 μL day^−1^: 1.5 μL, 3 μL, 4.5 μL, etc.). In the predator treatment, five individuals of the flatworm *Stenostomum virginianum* were added to each microcosm. *S. virginianum* is a voracious generalist predator known to prey on most ciliate species (Hammill, Kratina, et al., 2010; Núñez‐Ortiz et al., 2022). Preliminary tests showed that five individuals were enough to bring a population to extinction over a week.

The microcosms were monitored once every 3 h (~eight times per generation) for 4 weeks. The monitoring was performed by means of an automated system consisting of a camera (GXCAM HighChrome‐HR4 HI RES) connected to a stereomicroscope (Nikon SMZ1270) attached to a robotic gantry (igus drylin Gantry) programmed to record 12‐s videos of each microcosm every 3 h. All recorded videos were processed using ComTrack, an open‐source machine learning‐based software designed to extract individual morphological and behavioral information, as well as species abundances and spatial distributions of individuals from videos (Besson et al., 2021). Due to the impossibility of marking the individual ciliates in our system, data acquisition from videos is treated independently (i.e., we do not have repeated measures of morphology or speed across videos of the same individual). The accuracy of the software output in terms of abundance counts, morphological and movement measures was tested through additional experiments and crosschecked with the literature of the studied species (Besson et al., 2021; Hammill, Petchey, & Anholt, 2010; Hewett, 1988).

### Data processing

We processed the software outputs using R (version 4.3.1; R Core Team, 2022) to extract information on the speed and body length of every tracked individual of *P. caudatum* (see *Online data and code*), and the total number of individuals tracked (abundance) at every time point. From our raw dataset, we calculated the mean speed (^—^in millimeters per second)—as an indicator of behavior (Hammill, Kratina, et al., 2010)—and mean body (cell) length (in micrometers)—as a plastic morphological feature subject to variation in response to stressors (Uiterwaal et al., 2020)—of every individual in each of the 12‐s videos. Thus, across all the treatments and replicates, each tracked ciliate had a mean value for speed and length for each sampling point. For analysis, we averaged speed and body length values across all individuals and frames in each time point, resulting in a single feature value for each population per time point (Appendix S1: Figures S1–S3). On visual inspection, we found that the data were displaying a regular daily cycle of values throughout the study period, most evident in the abundance time series (Appendix S1: Figures S1–S3A). This occurred due to the daily topping up of each population with distilled water to maintain the initial volume: the mild perturbation of the microcosm (addition of the water) induced a sudden increase in movement and activity, and many of the motionless protists (not tracked in stable conditions) would become trackable, thus increasing the abundance counts. Due to the daily regularity of these spikes, we removed daily cycles in raw data using the additive seasonal trend decomposition by loess (STL, Cleveland et al., 1990). In the STL decomposition, we extracted seasonal components with a cycle of 8 observations (repeated every 9 observations to capture daily cycles). We subtracted the resulting seasonal time series from the raw data, resulting in a de‐cycled time series with trend and anomaly components. To confirm that abundance estimates reflected actual population sizes and not simply movement artifacts, we regularly inspected videos throughout the experiment and found that tracked counts closely corresponded to the visible number of ciliates present in each patch.

### Time series analysis

**FIGURE 1:**
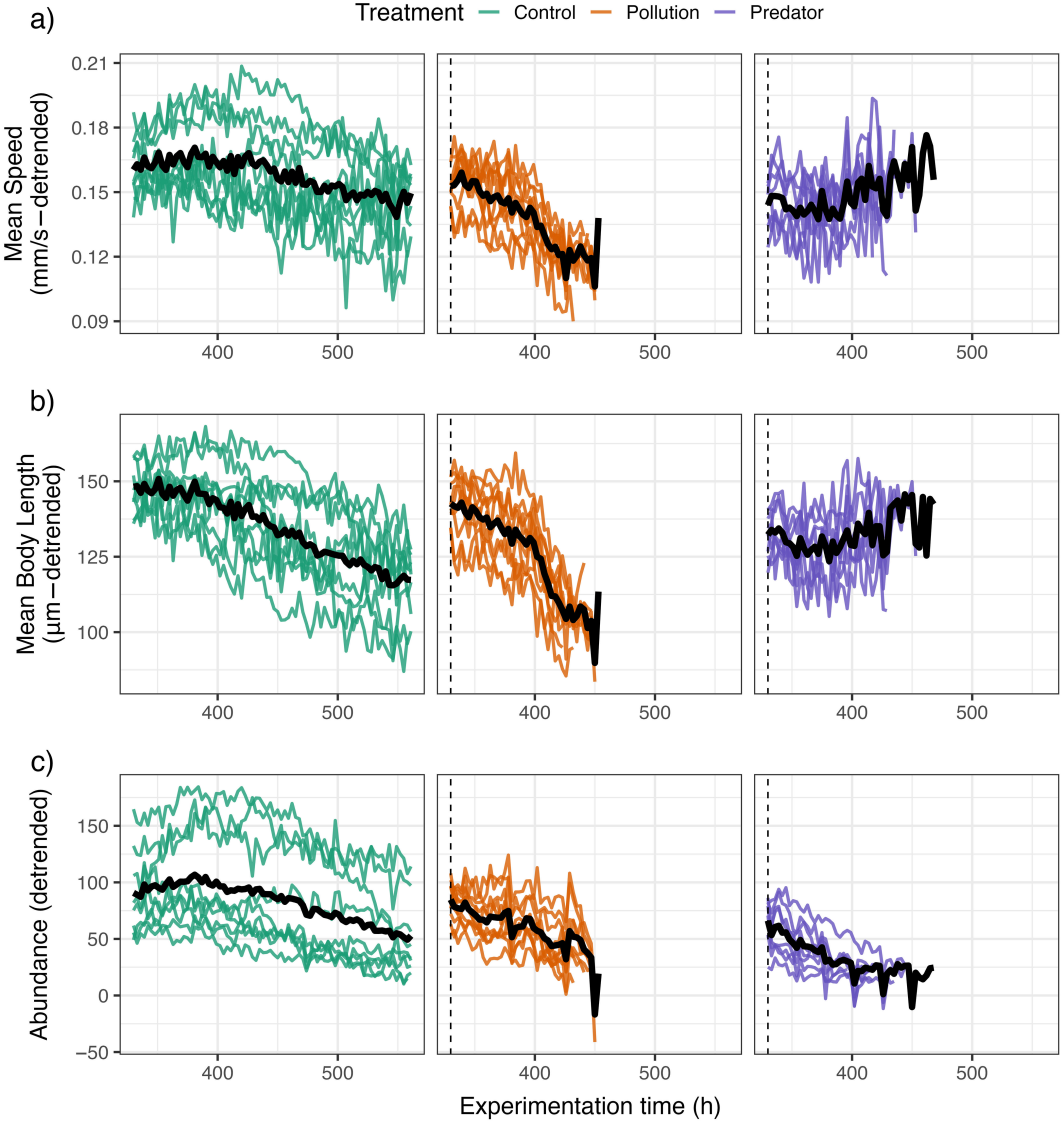
Detrended time series of the tracked *Paramecium caudatum* mean swimming speeds (a), body lengths (b), and number of individuals (c) in the three treatments (green—control, orange—pollution, purple—predator). Colored lines represent the single replicates. The thick black line is the mean of replicates. The dashed vertical line marks the start of the stressors.

We captured the temporal change in speed, length, and abundance (hereafter “components” of the timeline) using the de‐cycled data (Figure 1) and focused the analysis on the interval between the onset of the stressors (330 h after the beginning of the experiment) and the endpoint of the experiment (550 h, Figure 1). One replicate of the control treatment that collapsed for unknown reasons and two replicates of the predator treatment that did not collapse were excluded from the analysis (final *N* = 27). We removed the treatment replicates that did not undergo collapse to avoid conflating species trait changes that occur prior to collapse with those resulting from populations adapting to the stressor levels. Each time series in the stressor treatments was considered to reach “collapse” at the time point when the abundance fell below 10% (Aagaard et al., 2016) of the abundance after 10 days of growth and acclimation. To capture temporal changes in the components and pinpoint signals of the timeline, we used two analytical approaches that accounted for nonlinearity in temporal changes and differences in between‐replicate variability in the components. First, we used general additive mixed models (GAMMs) on each component in each treatment to identify the divergence point between stressed and control replicates. Second, we used a piecewise (or threshold) Bayesian regression fit across components in each treatment to estimate the relative timing of the component changes within a treatment. Full model descriptions are presented in Appendix S1: Section S1.

We fit GAMMs with the response variables of mean speed, mean length, and abundance of the populations across the three treatments (control, pollution, and predator). There was one model for each response variable in each treatment (to avoid pooling replicate‐level variation across timeline components/treatments), resulting in a total of nine statistical models. The key predictor of interest in the GAMMs was time point, to capture temporal trends in timeline components, which was included using both global‐ and replicate‐level smoothing terms (see Appendix S1: Section S1 for full details; Pedersen et al., 2019; Wood, 2003). GAMMs were fit using the *mgcv* and *nlme* packages (Pinheiro et al., 2017; Wood, 2011, 2017). After model fitting (see Appendix S1: Figures S5–S7), control‐ and treatment‐predicted time series were scaled linearly so that predicted values were 1 at the introduction of the stressor. This allowed us to improve the comparability of the treatments while maintaining raw units (in millimeters per second, in micrometers, and in number of individuals), as we focused on the mean temporal trends rather than on the values, while removing the differences present at the start of the stressors that were due to intrinsic variability of the individuals growth rate and phenotypic diversity across the treatments.

To test the robustness of the timeline signals sequence obtained using GAMMs, and more explicitly account for between‐replicate variance, we also performed time series analysis using piecewise or threshold regression (McClanahan et al., 2011; Roth et al., 2022), implemented in a Bayesian regression framework using the *brms* package (Bürkner, 2017). For piecewise regressions, we fit a single model for each treatment, modeling components together, which were *z*‐transformed for each replicate before analyses. For each model, we tested a linear regression with a single threshold (i.e., two linear slopes on either side of the threshold), which was chosen to reflect the impact of the stressor being added to the population. In each piecewise model, the linear coefficients on either side of the threshold, the intercept, and the threshold position (time point‐adjusting parameter) were estimated separately for each timeline component and each individual replicate. For full model and prior specifications, please see Appendix S1: Section S1.

### Early warning signals

To assess whether the smooth collapse of the populations was preceded by generic EWSs, we used the R package *EWSmethods* (O'Brien, Deb, Sidheekh, et al., 2023) to extract time series of the SD, CV, and lag‐1 autocorrelation for each replicate of each treatment, calculated with a rolling window approach using 50% of each time series (i.e., a temporal window starting with the first half of each time series). EWSs may be appropriate in this circumstance if the system displays a smooth transcritical transition (Drake & Griffen, 2010). Transcritical transitions differ from abrupt critical transitions in that the system responds smoothly (rather than abruptly) up to a fixed bifurcation point. This bifurcation point is not lost following transition, but the new state becomes stable and the old becomes unstable (O'Brien, Deb, Sidheekh, et al., 2023). For example, following extinction, a population cannot recover and so the old state (with positive abundance) is unstable. Conversely, critical transitions display hysteretic behavior where the system can recover following tipping (Clements & Ozgul, 2018).

We then fitted GAMMs on the time series of such indicators. The GAMMs had the same structure as described above (Appendix S1: Section S1). EWS components were not scaled for analysis because the CV and autocorrelation are scaled quantities. However, to improve comparability among replicates for EWS metrics, we modified the independent variable of time point to represent the hours before collapse; thus, every replicate EWS would end at the same time point (0).

### Pinpointing the timeline signals

Following our two statistical approaches for estimating temporal effects across timeline components, we estimated the timing of signals along the timeline in two ways. For GAMMs, we used a predictive, non‐Bayesian simulation framework, assessing the time points at which control and treatment populations diverged for each of the time series, and thus the occurrence of signals along the timeline. Specifically, we estimated divergence points as the time point at which CIs in the temporal trends of control and treatment populations no longer overlapped. We used “posterior simulation” to obtain robust CIs, which in this context is repeated simulations of predicted values using model coefficients, covariances, and their posterior uncertainty. We calculated predicted values using the multivariate normal distribution, making predictions of the temporal trend from each timeline component in each treatment, using 500 unique time‐point values between the start of the stressors and the end of the experiment. We made predictions including only the overall time‐point smoothing term coefficients, averaging over all additional coefficients and variation between replicates. Thus, predictions are for the mean temporal trend effect only. We sampled 1000 unique values of temporal trend coefficients (basis dimension coefficients from the smoother term) under parameter uncertainty using a multivariate normal distribution (implemented in the MASS package; Venables & Ripley, 2002). We combined simulated model coefficients with the linear prediction matrix to retrieve 1000 sets of predicted values. Then, across the resultant 1000 sets of predictions, we calculated the upper and lower 95% CLs of the temporal trend. We defined the divergence point between control and treatment predictions as the last point at which the CI of the control overlapped with the treatment prediction, that is, the time point where the difference between the upper and lower CIs of control and treatment reached 0. In the results, we refer to such cases as the variables showing “significant” change. Finally, we assessed the sensitivity of the divergence points to the posterior resampling: we resampled 10% of the predicted data and recalculated 100 divergence points from the bootstrapped time series, to give an estimate of divergence uncertainty.

For piecewise regression models, divergence points were estimated for each replicate individually by estimating the threshold parameter (time point at which linear trend in timeline component changed), which we sampled with draws of the posterior distribution. We used these two methods to estimate divergence points to test the robustness of timeline signals sequence and to explore the implications of explicitly estimating divergences for each replicate individually, accounting for among‐replicate variance.

## RESULTS

We observed some variability among the replicates of control populations, with one undergoing collapse spontaneously (not shown in Figure 1), and three replicates gaining higher densities compared to the remaining six, which were similar in their abundance trends (Figure 1). All polluted populations underwent collapse after approximately five generations from the initial introduction of the stressor (Figure 1).

**FIGURE 2:**
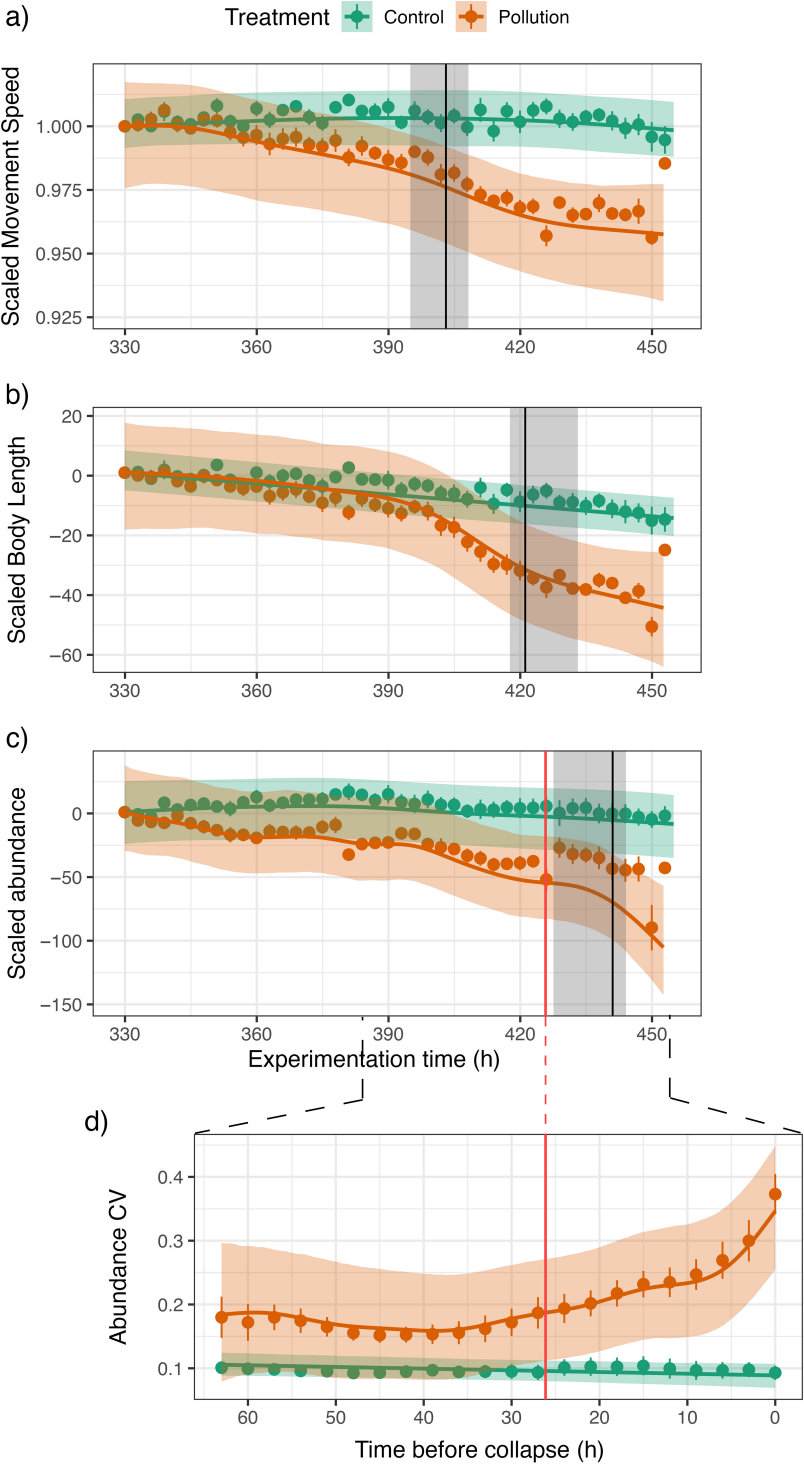
Observed timeline to collapse for the pollution treatment. Detrended and scaled (see Appendix S1) time series of mean swimming speeds (a), mean body lengths (b), abundance (c), and CV (zoomed panel d) starting from the beginning of the stressor treatment (time point 330). The CV is calculated on 50% of the abundance time series and the *x* axis is converted into hours before collapse to normalize the temporal component for each replicate (i.e., all time series end at the same time point) (d). Orange and green elements represent the pollution and control treatments, respectively. Dots with lines represent across‐replicate mean and SEs. Colored lines and areas represent predicted (using general additive mixed models [GAMMs]) mean trends and 95% CIs, respectively (a–d). For analysis, predicted time series of control and pollution treatments were scaled linearly to begin at a value of 1 when the stressor was introduced. Vertical black lines and gray areas indicate the divergence time point of the CI trends between the two treatments (a significant change compared to the control) with a posterior resampling interval. The red vertical line in (c) and (d) indicates the emergence of an early warning signal (EWS) (a significant difference in the CV between the pollution and the control treatment), also projected on the abundance panel.

**FIGURE 3:**
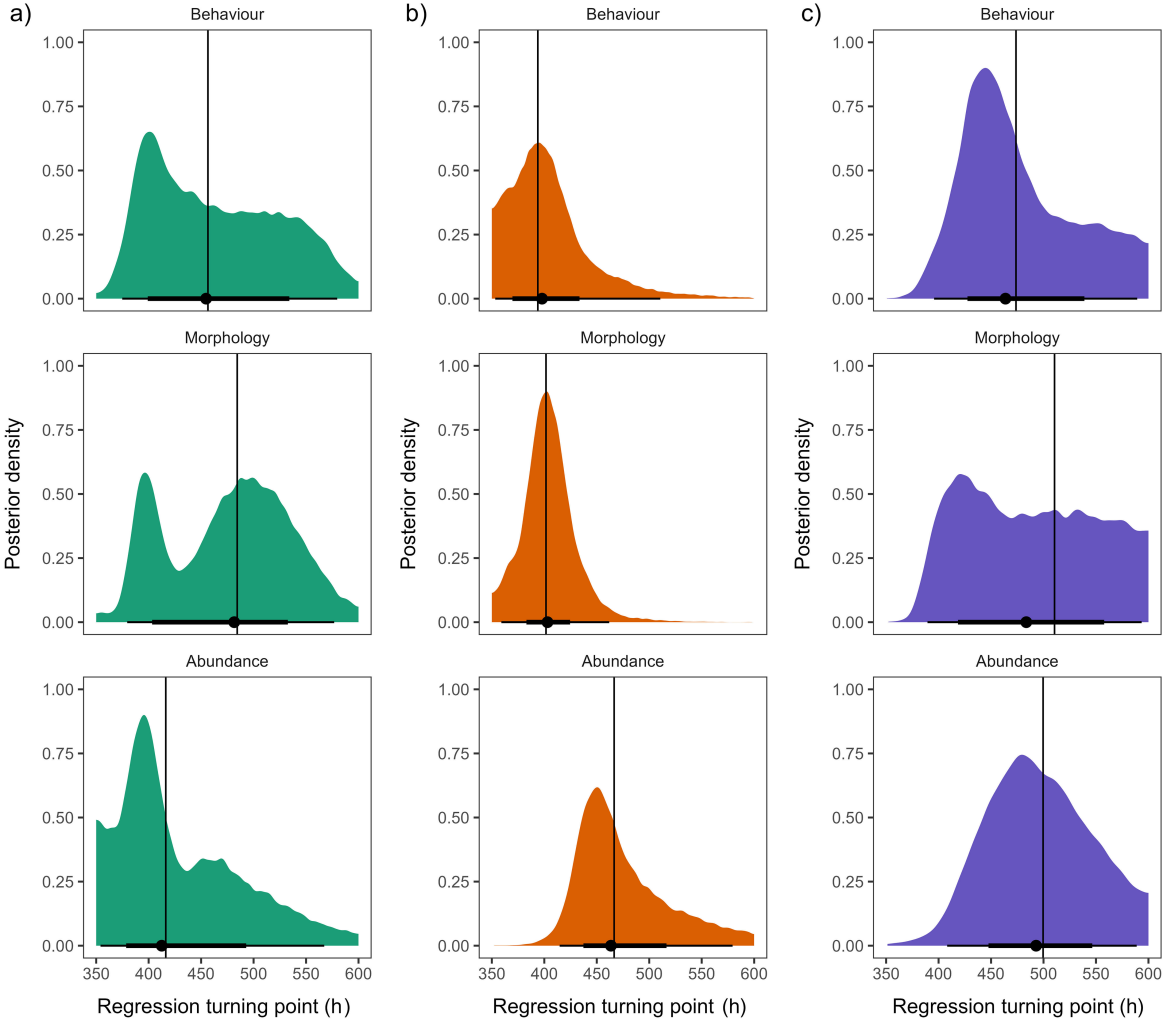
Posterior density for threshold time points across timeline components in the control (a), pollution (b), and predator (c) treatment from piecewise regression models. Density distributions give the full posterior density of the threshold parameter, scaled to time in hours, across replicates for each of the timeline components. Segments give the credible intervals based on the Cumulative Distribution Function for each timeline component, and vertical lines give the 50% quantile of the posterior distribution.

We found evidence for a timeline to collapse in the pollution treatment, which increased the forecast horizon relative to EWS analysis (Figures 2 and 3). From overall temporal trends estimated from additive models, the mean movement speed significantly reduced after 3.18 generations from the start of the stressor (73 h [65; 78], upper and lower 95% CLs of divergence point based on posterior resampling), when compared to the control treatments (Figure 2a). Then, 3.96 generations (91 h [88; 103]) after the beginning of the pollution, and 0.78 generations (18 h) after the behavioral change, the mean body length began to decline more strongly than in the control populations (Figure 2b). Finally, 4.83 generations (111 h [98; 114]) after introducing pollution, and 0.87 (~20 h) generations after the morphology signal, the abundance trend diverged significantly from the control. The predicted abundance trend reached the point of collapse at time point 451 h, that is, the ramping pollution brought the population to functional extinction after 5.34 generations, and the behavioral and morphological shifts preceded the collapse by 2.1 and 1.3 generations, respectively. The EWS analysis revealed a significant divergence in abundance CV 1.13 generations (26 h) before the collapse point, but after changes in both the behavior and morphology (Figure 2). Critically, the introduction of behavioral and morphological data increased the forecast horizon of population collapse by one generation relative to the EWS analysis. Finally, the observed divergence points were robust to posterior resampling of predicted temporal trends, displaying a consistent sequence of change in behavior, morphology, and abundance (preceded by an increase in abundance CV, Figure 2).

We obtained qualitatively similar results from piecewise regression models for pollution (Figure 3b), for which behavioral and morphological time series shifted 3.17 and 2.83 generations before abundance across replicates, respectively. However, behavioral and morphological shifts were estimated to occur closer together in time than in GAMMs. In the piecewise models, the posterior median threshold for behavioral time series change across replicates was 2.78 generations (63.9 h [39.6; 87.8]) following the introduction of the stressor, with a morphological shift immediately after at 3.12 generations (71.7 h [56.2; 86.7]). Then, the threshold for abundance time series change was 5.95 generations (137 h [115; 179]) following the stressor introduction. Together, results from additive and threshold models indicate a clear sequence of timeline signals in the pollution treatment.

**FIGURE 4:**
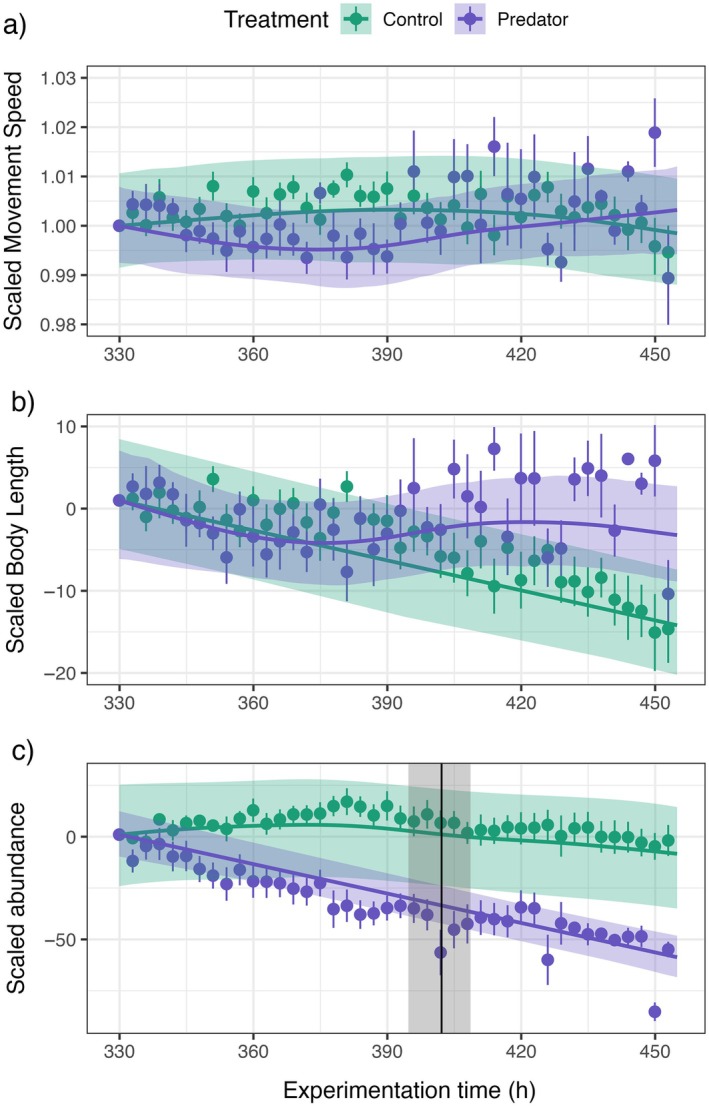
Detrended and scaled time series of mean swimming speeds (a), mean body lengths (b), and abundance (c) starting from the beginning of the stressor treatment. Elements in purple represent the predator treatment, green elements the control treatment. Dots with lines represent across‐replicate means and SEs. Continuous lines and gray areas represent the general additive mixed models (GAMMs) predicted mean trends with 95% CIs. (a–c) For analysis, predicted time series of control and pollution treatments were scaled linearly to begin at a value of 1 when the stressor was introduced. Vertical black lines and gray shaded areas indicate the divergence time point of the CI trends between the two treatments (a significant change compared to the control) with a posterior resampling interval.

In contrast to the pollution treatment, the introduction of the flatworm predator was not followed by sequential changes in behavior, morphology, and abundance, both in GAMMs (Figure 4) and in piecewise regressions (Figure 3c). Instead, a decline in the abundance of populations in the predator treatment began immediately following the introduction of the stressor, with a significant deviation in predicted abundance after 72 h [64; 78] or 3.14 generations (Figure 4c). We found no clear trends of deviation in mean speed and body length across the predator experiment (Figure 4a,b), and this resulted in the wide intervals for turning points in the posterior density (Figure 3c). For the control treatment, no pattern in timeline signals was identified from the piecewise regressions (Figure 3a).

We also assessed temporal trends in other abundance EWS metrics (SD, and lag‐1 autocorrelation—ACF; Appendix S1: Figure S8), normalizing the temporal component for each replicate using the time before collapse (time before experiment end for control treatments). The EWS analysis did not show clear patterns in the predator treatment; the CV was highly variable and displayed an increasing trend deviating significantly from the control since the addition of predators (Appendix S1: Figure S8).

## DISCUSSION

The rapid pace of change in the natural environment is altering the dynamics of wild populations (Capdevila et al., 2022), and EWSs of population collapse are poised as a crucial tool for biodiversity management and protection (Nijp et al., 2019; Stelzer et al., 2021). However, the application of these methods is currently hampered by their reliability and their limited forecast horizon (Baruah et al., 2020; Patterson et al., 2021). Expanding these predictive frameworks to include information at the individual level gives rise to potentially powerful approaches to forecast population collapse (Clements et al., 2017; Clements & Ozgul, 2016a). Similarly, new conceptual work suggests that the temporal sequence of changes in individual and population traits gives rise to a corroborative tool to infer risk of population collapse and trigger preemptive conservation actions (Cerini, Childs, & Clements, 2023; Keen et al., 2021; Pirotta et al., 2018). Here, using a novel autonomous, high‐throughput monitoring system allowing us to track individual behavior, morphology, and population abundance over 24 generations, we found experimental evidence to support the conceptual model of a timeline to population collapse (Cerini, Childs, & Clements, 2023). Namely, we find a sequential shift in behavior, then morphology, and finally abundance in response to a gradually increasing environmental stressor. Such signals were detectable up to 1.65 generations before a significant decrease in abundance (i.e., a statistically supported decline compared to the control treatment), and crucially a whole generation before a detectable EWS. The successive changes from the individual to the population level represented a growing body of evidence, strengthening the inference of an approaching collapse. However, the occurrence of the timeline to collapse is dependent on the stressor nature. We highlight that integrative ecological monitoring approaches, with an emphasis on individual behavioral and morphological data in addition to population monitoring, may represent a crucial next step to anticipate population declines more effectively.

The environmental pollution stressor generated a clear timeline of changes matching the theoretical framework (Cerini, Childs, & Clements, 2023). Generally, changes in behavior are among the earliest and commonest responses to environmental change; stress‐induced behavioral change can take the form of shifts in activity patterns, distributional range, or ecological choices (e.g., antipredatory behaviors, foraging, nesting, or reproductive preferences; Berger‐Tal et al., 2011; Rabaiotti & Woodroffe, 2019). In our experiment, the behavioral shift preceded the morphological shift by nearly a whole generation if we consider the GAMM results, indicating that the locomotory system of *P. caudatum* was the first to be affected by the pollutant. Copper ions are known to affect the food vacuole‐forming capacity and chemotaxis in ciliates (Dale, 1991; Nilsson, 1981). Thus, the observed decrease in movement speed was likely due to a general energy availability reduction for the *P. caudatum* cells, as their food intake system was compromised. Often, behavioral traits are more plastic than other features (e.g., life history; Refsnider & Janzen, 2012), and can buffer organisms against resource reduction (Goossens et al., 2020) or temperature change (Chen et al., 2011). After the shift in cell movement could not avert a physiological change, there was a reduction in food intake affecting *P. caudatum* morphology. The timing between shifts in individual traits will vary between species, but a behavioral change may occur up to and over a generation time before morphological change. For example, food resource reduction increases the foraging distance in seabirds (Fayet et al., 2021). In this case, we might observe a longer time gap before morphology and abundance are affected. Ultimately, however, characterizing individual phenotypic traits can add important insights into population decline (Cerini, Childs, & Clements, 2023; Tyack et al., 2022).

The next observed signal in the timeline was the shift in morphology. Change in morphological features is a general physiological response not only to energy intake reduction (e.g., mass reduction) but also to other kinds of stressors such as environmental warming (Sheridan & Bickford, 2011) and predator presence (Chiba, 2007). The mean *P. caudatum* length declined strongly before the trend nearly plateaued over an approximate two‐generation time interval (Figures 1 and 2). At that point, most individuals were likely unable to replicate, properly eat, and digest, and the population abundance started to decrease. Generally, the observation of a morphological shift shortly after, or concurrently with, a behavior change should be considered a pivotal moment in view of management of vulnerable populations, as it may be the last signal observable before stressors act directly on the survival of individuals (i.e., affecting the population dynamics; Cerini, Childs, & Clements, 2023). Indeed, for larger organisms such as vertebrates, the capacity of body size to change for long periods of time (e.g., polar bears weight loss; Stirling & Derocher, 2012) as a reaction to stressors provides an opportunity to perform conservation actions.

After the morphological change, the populations showed a rapid decline to collapse in little more than one generation. Before collapse, we observed a potential EWS: an increase in the CV of abundance, diverging from the control approximately one generation before collapse. This might represent a final measurable signal in the timeline before the decline in abundance, becoming visible after the behavioral and morphological change. The CV proved to be a useful EWS forecasting abrupt changes in other systems (Clements & Ozgul, 2018). However, the CV was one indicator out of three tested (see Appendix S1: Figure S4), and EWS metrics are prone to indicate false positives (Boettiger & Hastings, 2012; Burthe et al., 2016). Additionally, despite the populations undergoing a steep decline to collapse, they are not necessarily undergoing a form of transition that EWS can detect (O'Brien, Deb, Gal, et al., 2023). Thus, solely relying on EWS metrics may not be a robust approach. Indeed, EWSs were only generated ~ one generation prior to collapse (half the forecast window of the first behavioral signals), and thus may well be insufficient for meaningful action in a real‐world conservation scenario. Hence, the observation of the timeline signals before the EWS builds not only a wider predictive horizon but also acts as a quality check for classic collapse indicators. By confirming that EWSs, if observed after the occurrence of the other signals, are actually representing imminent collapse, the timeline framework can help in reducing the false‐positive rates of classic EWSs. In fact, if behavioral or morphological change can also show false positives (e.g., plasticity response), their successive temporal sequence represents a clear indication that all the population's stress‐coping mechanisms are being involved, and thus the population might be at risk of collapse if pressure keeps building (Cerini, Childs, & Clements, 2023).

In contrast to the pollution treatment, the predator introduction, a press disturbance scenario, did not result in the predicted sequence of observable events before the population collapsed. While ciliates, and the *Paramecium* genus in particular, are known to display antipredatory behavioral and morphological responses (Cerini, O'Brien, et al., 2023; Fyda et al., 2005), neither behavioral nor morphological traits showed a clear pattern of change compared to the control in the predator treatment. Exposure to predatory *Stenostomum* cues induced a swimming speed reduction in *Paramecium* and a cell shape change toward a ball‐shaped morph (to lower the risk of being eaten by the predator; Hammill, Petchey, & Anholt, 2010). Our lack of observation of similar responses may be a function of the predation pressure being too high, and the size of the habitats too small, for the *P. caudatum* individuals to escape or implement antipredatory responses. Thus, predation had an immediate impact on the abundances of the ciliate populations, potentially reducing the opportunity to elicit measurable behavioral or morphological change. Such contrast between responses to predators and pollution supports previous work suggesting that the rapid onset of high‐intensity stressors (e.g., extreme events) can prevent meaningful prediction in wildlife populations (Clements & Ozgul, 2016b) and suggests that ramp disturbances (environmental pollution, temperature increase, habitat loss) are the best‐suited scenario for the timeline application. Additionally, this suggests that the observation of the timeline to collapse will depend on the mechanisms by which the stressor acts on the population.

Adapting the timeline to collapse approach we used here for wildlife conservation is challenging, particularly due to the absence of standard controls and the nondeterministic responses of populations to stressors. These challenges were evident in our controlled experimental system and are expected to be more pronounced in field studies. On the one hand, the use of historical thresholds (Donadio Linares, 2022) or comparisons with nonstressed populations (space‐per‐time substitution; Fayet et al., 2021) can help in pinpointing significant deviations in the behavioral or morphological traits of studied populations, following our GAMM approach. However, even our control populations exhibited instability, with each replicate displaying unique dynamics despite standardized treatments (Figure 1). This underscores the limitation of applying the timeline framework with GAMMs to aggregated time series without a clear reference population and highlights the necessity of incorporating population‐level variability into timeline comparisons. We addressed this by allowing our GAMMs to vary by replicate around a global trend, enabling us to capture both overall patterns and replicate‐specific sequences of change. Additionally, we compared the observed sequence with that from the piecewise regression, which does not require a control treatment comparison. Integrating both methods could strengthen our ability to detect early signals of collapse in complex ecological systems.

Regardless of the method, concurrent monitoring of individual traits and abundance has the potential to increase the time window available to implement management actions, and thus developing frameworks to leverage multidimensional data remains a priority. For example, had we limited ourselves to monitoring swimming speed of the ciliates, we might have wrongly interpreted the observed change in speed as a new behavioral optimum of the individuals that are responding to the increasing pollution; in fact, after a swift reduction, the average swimming speed approached a possible plateau (Figures 1 and 2a after time point 420) before the abundance decline. The new value might have been the physiological plastic limit of that behavior, as at that point the pollution concentration was already critical and started to affect individuals' survival right after (i.e., abundance continuously declines). In such case, managing actions (i.e., cutting a chemical waste from an ecosystem; Vorobeichik, 2022) might be useless. Instead, focusing on the temporal sequence of multiple signals reduces the risk of wrongly considering the population as adapting to the new environmental conditions. In fact, if observing a behavioral change might flag a situation of potential future risk and thus should trigger investigations on possible stressors acting on the population, observing a morphological change after the behavioral one should be considered a critical moment for implementing management actions. Projecting our experimental example in the real world, and considering aquatic organisms with longer generation times (e.g., fish), after the observation of the first behavioral signal a practitioner might have started analyzing environmental variables potentially identifying the chemical stressors; the body size change signal would have confirmed that the stressor level is reaching dangerous levels, but there would still be a generation's time before the abundance starts to decline, to stop the pollution, ultimately avoiding the population collapse.

More experiments are needed to increase both the spectrum of tested stressors and the complexity of the taxa. Mesocosm experiments with multicellular organisms whereby one can measure also detailed demographic processes like fecundity are the obvious next step to test the other scenarios where the framework is best suited. Nevertheless, we urge ecologists to take advantage of autonomous monitoring tools for biodiversity monitoring (Besson et al., 2022; Cavender‐Bares et al., 2022), which can collect multidimensional data to more effectively predict population change, with few disadvantages (e.g., costs, proper calibration). Ultimately, the goal of the timeline to collapse is to fuel the implementation of the multidimensional approach directly into predictive frameworks, to add to a large repertoire of forecasting tools and EWSs using a wider array of data typologies. Although this aspect is not the focus of our paper, we believe that generating real‐world data on collapse dynamics, with their potential idiosyncrasies, is a useful first step in that direction.

In conclusion, our results demonstrate a timeline of signals preceding population collapse (Cerini, Childs, & Clements, 2023), which can improve the reliability of EWSs and, critically, flag situations of potential risk to conservation practitioners much earlier than if considering only population‐level dynamics. A ramping pollution stressor represents a clear case where the individual traits displayed shifts more than two generations before a decline in abundance. Additionally, the observation of the sequence of signals indicates that as the stressor is increasing, different dimensions of a population are being engaged, thus giving insights on the adaptability, or lack of it, of the population to new conditions.

## AUTHOR CONTRIBUTIONS

Christopher F. Clements and Dylan Z. Childs formulated the initial theoretical framework. All authors aided in experimental design. Francesco Cerini performed the experiments. Francesco Cerini and John Jackson performed data wrangling and analysis. Duncan O'Brien provided expertise and support for the analysis and exploration of different methodologies. Francesco Cerini and John Jackson wrote the first draft of the manuscript. All authors contributed equally to the final draft. Both Francesco Cerini and John Jackson are to be considered first authors.

## CONFLICT OF INTEREST STATEMENT

The authors declare no conflicts of interest.

## Supporting information


Appendix S1.

## Data Availability

Data and code (Jackson, 2025) are available in Zenodo at https://doi.org/10.5281/zenodo.14833814.
