# Supplementary material for: Multivariate signals of population collapse in a high‐throughput ecological experiment
Source: Ecology. 2025 Sep 15;106(9):e70197. doi: 10.1002/ecy.70197 (PMC12436010; doi:10.1002/ecy.70197)
Supplement: Supplementary file 1 — Appendix S1. [file ECY-106-e70197-s001.pdf]

Appendix S1

Ecology

Multivariate signals of population collapse in a high-throughput ecological experiment

Francesco Cerini, John Jackson, Duncan O’Brien, Dylan Z. Childs, Christopher F. Clements

Section S1. Model specifications

Our first set of models to test the occurrence of timeline signals were generalised additive mixed models (GAMMs), which we fit for each treatment in each timeline component (mean speed, mean length, and abundance) separately. The general form of the model was as follows, where the mean timeline response is variable τ, for row i (a replicate population in a given time step) and replicate r,

$$\tau_{i,r} = \alpha + f_t(T_i) + f_R(T_{i,r}) + U_r + \beta_a G_i,$$

where α is the global intercept. The term $f_t(T_i)$ is the key overall predictor of time point (T), for which the smoothing function f_t was fit with a thin plate regression spline (Wood 2003). The basis dimension of $f_t(T_i)$ for each response variable was determined using AIC model selection for a range of k values (Fig. S5). We also included $f_R(T_{i,r})$, which gives individual time-point smoothing terms for each replicate population, to account for the variability in temporal patterns between replicates following Pedersen *et al.* 2019. Individual smoothing terms given by f_R are analogous to interaction effects or random slopes, with an overall temporal smoothed effect that is independent for each grouping variable. We fit a general random effect term, U_r, for replicate populations, to account for intercept differences between replicates (Wood 2017). Separately, we explored patterns of temporal autocorrelation in each of the time series using partial autocorrelation, and determined that lag-1 autocorrelation terms were sufficient in each of the three response variables (Fig. S6). Therefore, we also included the term $\beta_a G_i$, where G gives the time point expressed as a categorical factor, and the coefficient β_a gives a lag-1 autocorrelation coefficient fit using the *nlme* package from within *mgcv* in R (Pinheiro *et al.* 2017). All models were fit with a Gaussian distribution, and we assessed the appropriateness of Gaussian models by visually assessing frequency histograms for each response variable, and diagnostic plots of model residuals against fitted values for each additive model (Fig. S4; Fig. S7). After model fitting, control and treatment predicted timeseries were scaled linearly so that predicted values were 1 at the introduction of the stressor. This allowed us to improve the comparability of the treatments while maintaining raw units (mm/s, µm, and number of individuals), as we focused on the mean temporal trends rather than on the values, while removing the differences

present at the start of the stressors that were due to intrinsic variability of the individuals growth rate and phenotypic diversity across the treatments.

Our second approach to testing the occurrence of a sequence in timeline signals was using piecewise linear regression models fit using a Bayesian regression, for which we used a single threshold model that assumed a linear relationship through time either side of a threshold point (McClanahan *et al.* 2011; Roth *et al.* 2022). Piecewise regressions were fit for each treatment only, and we explicitly modelled the different components of the timeline (swimming speed, body length and population abundance time series) and replicate-level variation within the same models. In this case, response variables were z-transformed for each replicate in each component prior to analysis to allow for comparability between timeline components within the same model. The full model structure and prior specifications are given by:

$$\tau_i \sim Normal(\mu_i, \sigma)$$

$$\mu_i = K_{0,i} + K_{1,i} \cdot \frac{T_i - \omega_i}{1 + e^{-(\omega_i - T_i)}} + K_{1,i} \cdot \frac{T_i - \omega_i}{1 + e^{-(T_i - \omega_i)}}$$

$$\omega_i = \frac{T_{max,i}}{e^{-(\alpha_i)}} + T_{min,i}$$

$$K_{0,i} = \beta_{1,c,i} + \beta_{2,c,r,i}$$

$$K_{1,i} = \beta_{3,c,i} + \beta_{4,c,r,i}$$

$$K_{2,i} = \beta_{5,c,i} + \beta_{6,c,r,i}$$

$$\alpha_i = \beta_{7,c,i} + \beta_{8,c,r,i}$$

$$\beta_1, \beta_3, \beta_5 5, \beta_7 \sim Normal(0,0.2)$$

$$\beta_2 \sim Normal(0, \sigma_2)$$

$$\beta_4 \sim Normal(0, \sigma_4)$$

$$\beta_6 \sim Normal(0, \sigma_6)$$

$$\beta_8 \sim Normal(0, \sigma_8)$$

$$\sigma \sim exponential(5)$$

$$\sigma_2, \sigma_4, \sigma_6, \sigma_8 \sim student\_t(3,0,10)$$

where the response variable (z-transformed timeline components in each treatment), τ, was given by a Gaussian distribution. The core model was specified with three parts. First, the global intercept of the timeseries model, K_0, was estimated for each timeline component, c, separately, which was specified with a fixed effect, and for each replicate, r, separately, which was specified as an intercept-only random effect. Then, the linear coefficient for the relationship between timepoint, T, and the response variable before the threshold point was given by β_1, which was estimated for each timeline component and for each replicate separately as above. We enabled for a smoothed change in the response variable around the threshold using an inverse logit relationship. The threshold point was given by the control parameter ω, which was also specified for each timeline component and each replicate separately. The threshold parameter was scaled to the time window of an individual replicate using a second inverse logit equation with control parameter α, which was specific to the start and end time points for each replicate timeseries. Finally, the linear coefficient for the relationship between timepoint, T, and the response variable after the threshold point was given by β_2, which was estimated for each timeline component and for each replicate separately, and controlled using an inverse logit threshold point determined by ω, as above. The β coefficients and control parameter α were estimated using weakly informed Gaussian priors, and the population level standard deviation σ was estimated with an exponential prior. Models were run over three Markov chains for 3000 iterations (including 1500 warm up iterations), and we assessed model convergence by assessing $\hat{R}$ hat values, which estimate the degree of mixing between chains. For full specification please see the Online code at Zenodo repository DOI: 10.5281/zenodo.10160252.

Figure S1. Raw time series (column A) and detrended time series (column B) of the *Paramecium caudatum* mean swimming speed, mean body length and abundance of each replicate (N = 10) in the Control treatment.

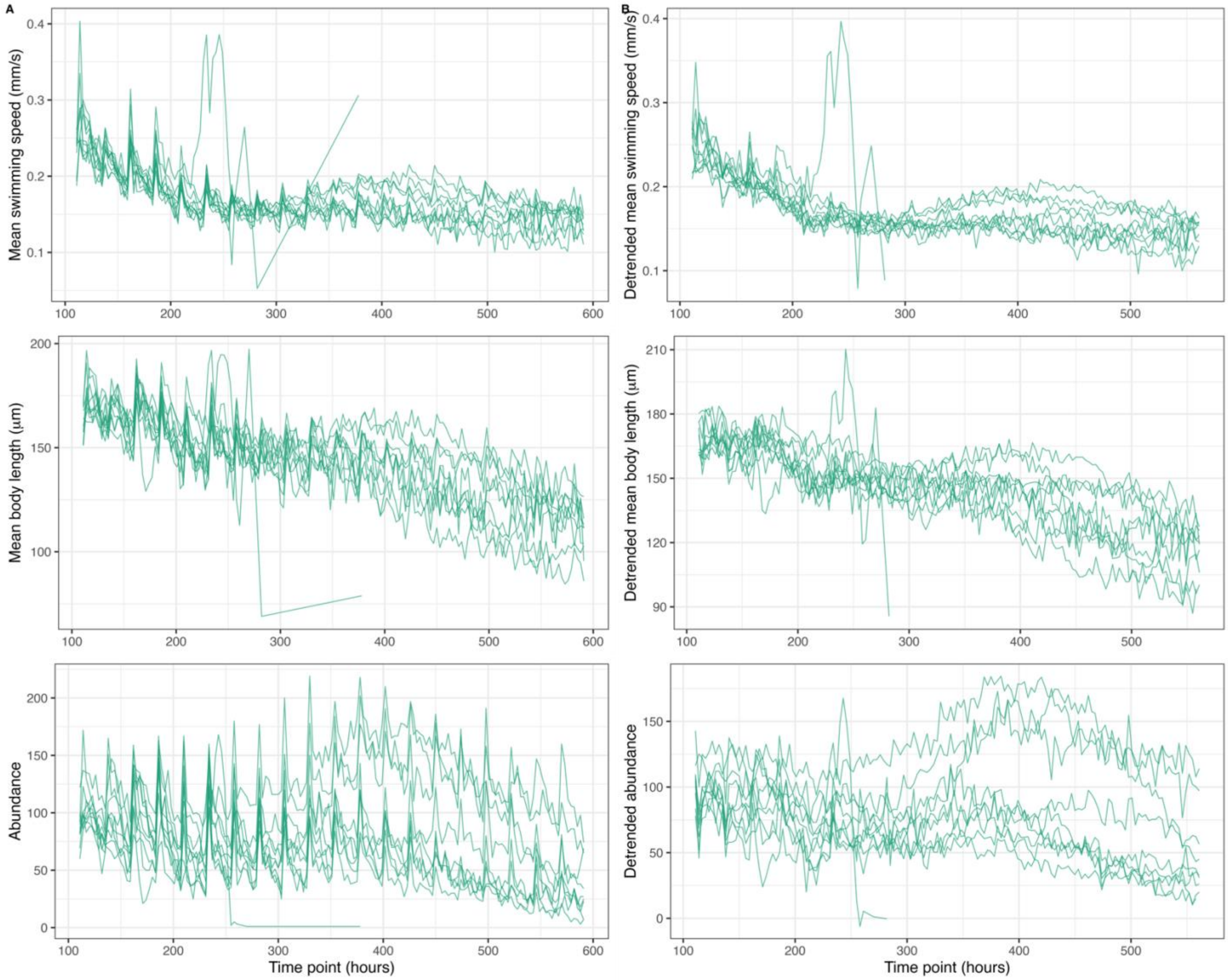

Figure S2. Raw time series (column A) and detrended time series (column B) of the *Paramecium caudatum* mean swimming speed, mean body length and abundance of each replicate (N = 10) in the Pollution treatment. The red dashed line marks the beginning of the stressor (daily addition of a growing Cu^{2+} quantity).

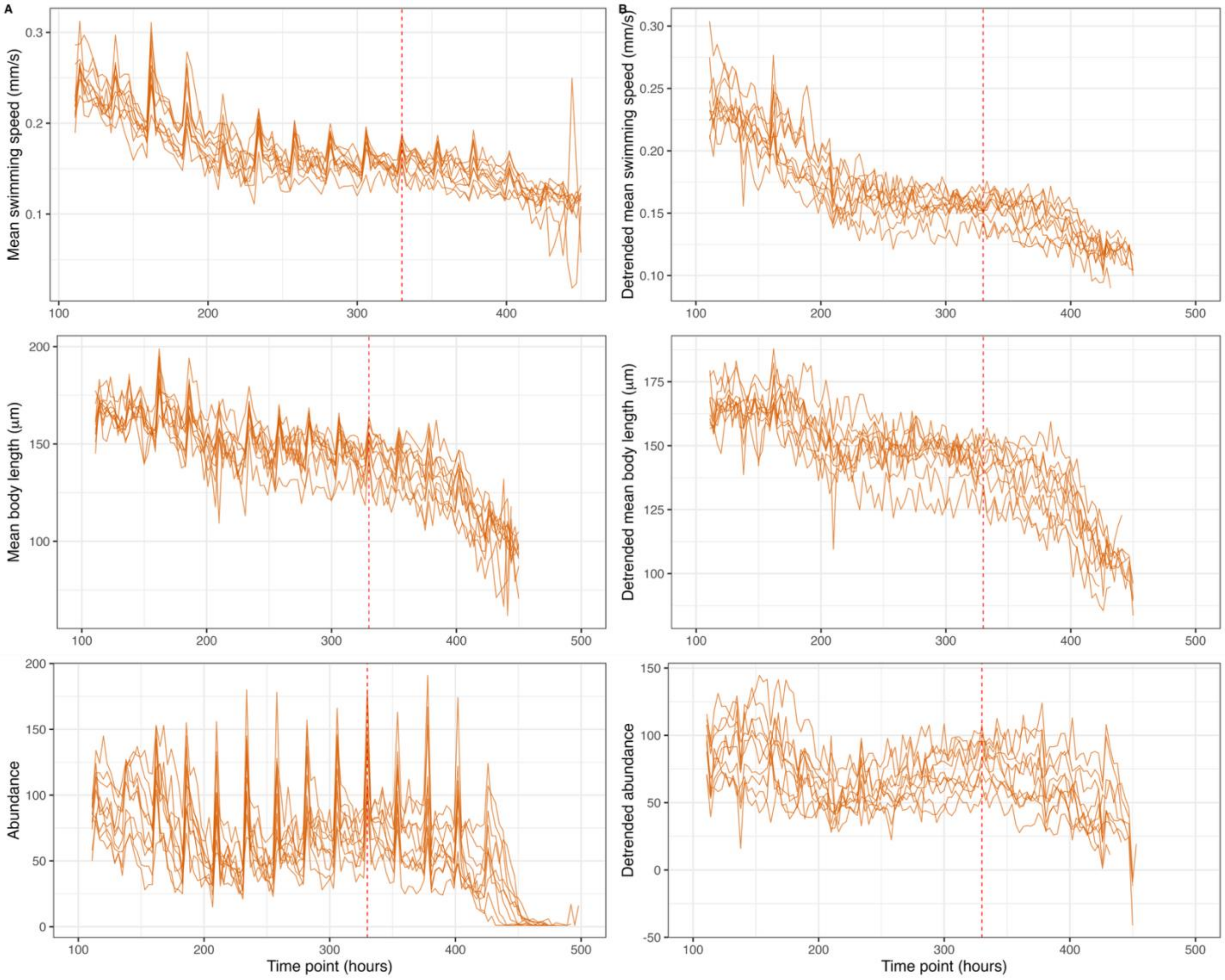

Figure S3. Raw time series (column A) and detrended time series (column B) of the *Paramecium caudatum* mean swimming speed, mean body length and abundance of each replicate (N = 10) in the Predator treatment. The red dashed line marks the beginning of the stressor (introduction of 5 individuals of the predator flatworm *Stenostomum virginianum*).

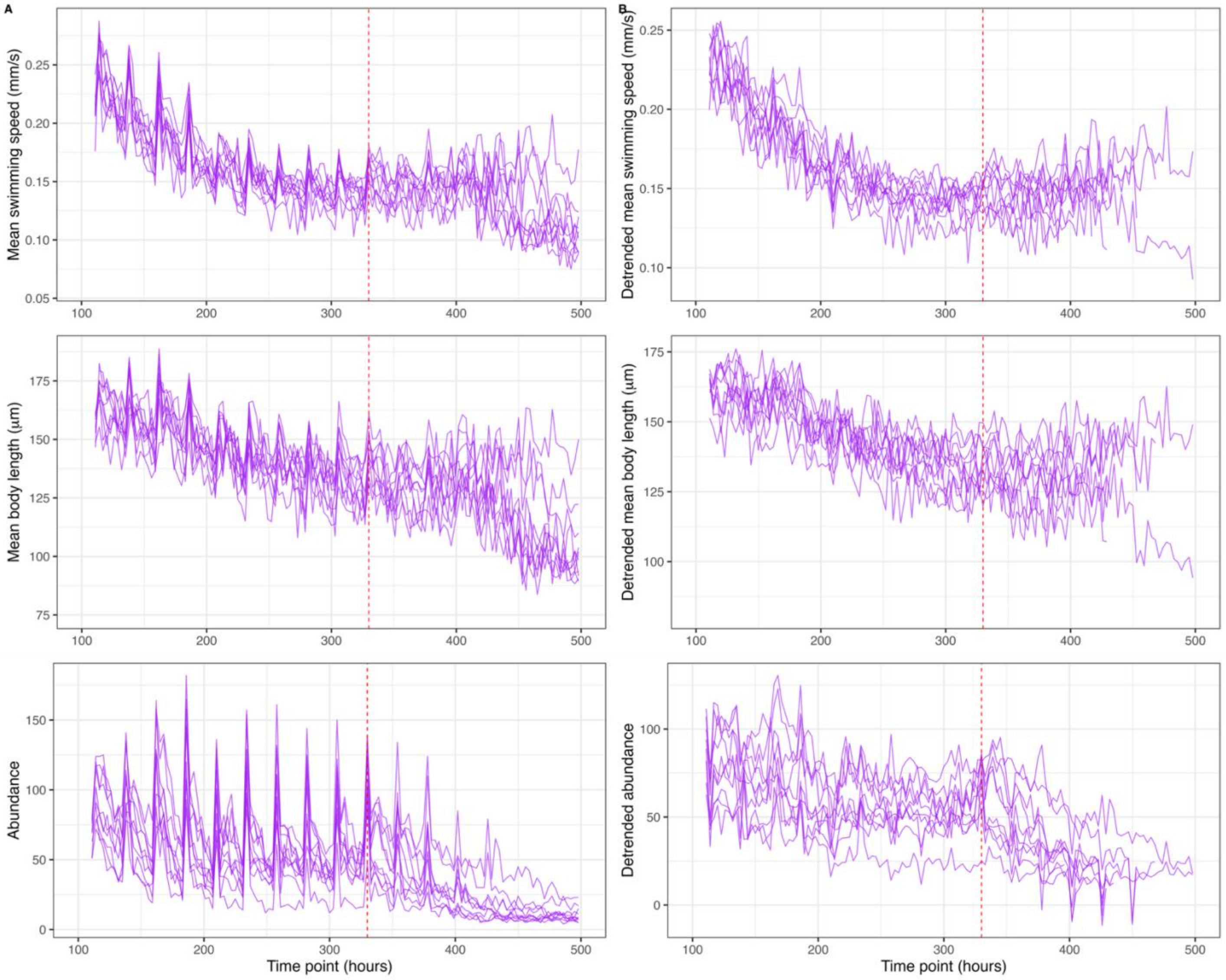

Figure S4. Frequency histograms of raw, detrended timeline components (movement speed, body length, and abundance), used in additive models. Colour denotes treatment, with frequency observations stacked by treatment.

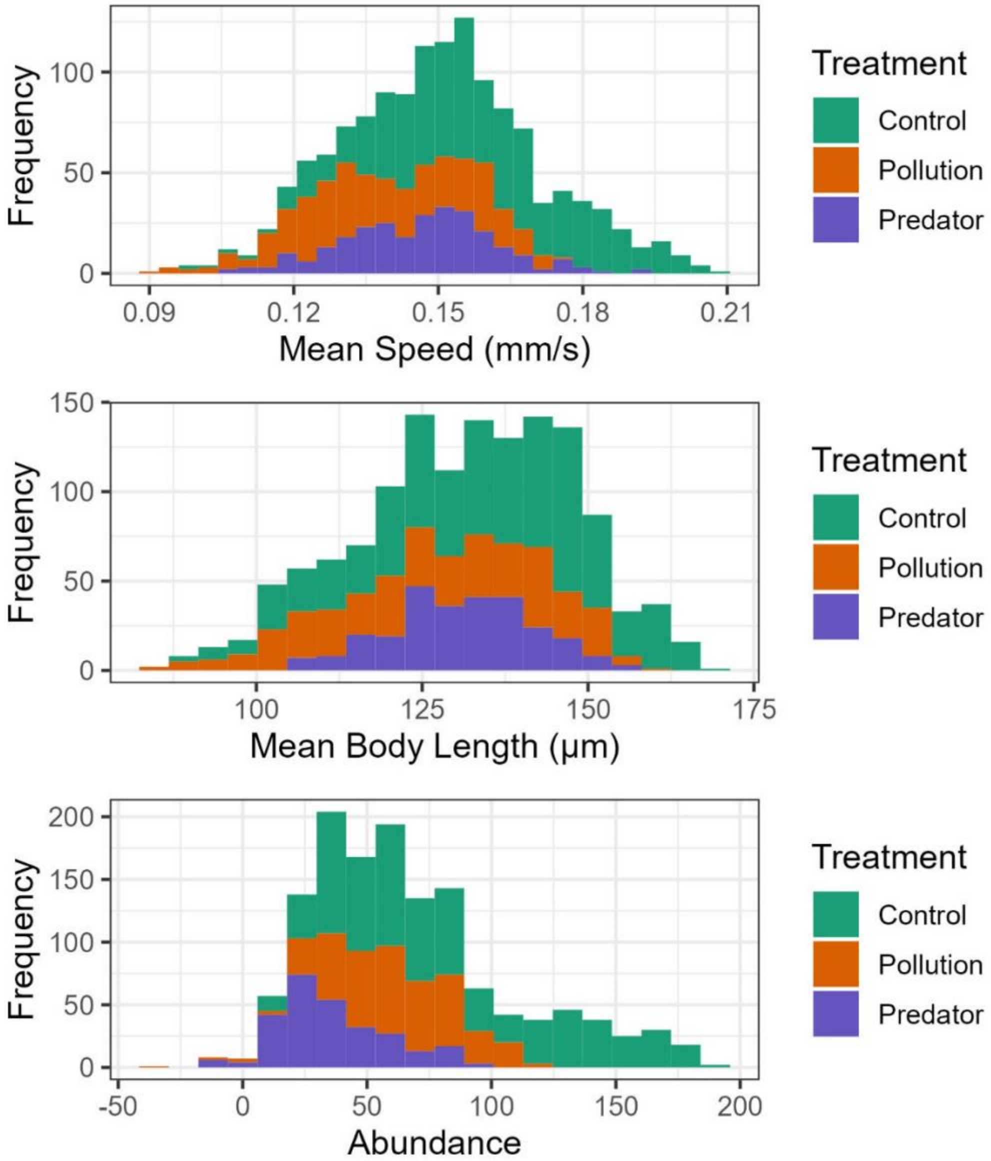

Figure S5. Model selection for basis dimension (k) in additive models. The basis dimension values selected were 17, 11, and 12, for abundance, body size and movement speed, respectively.

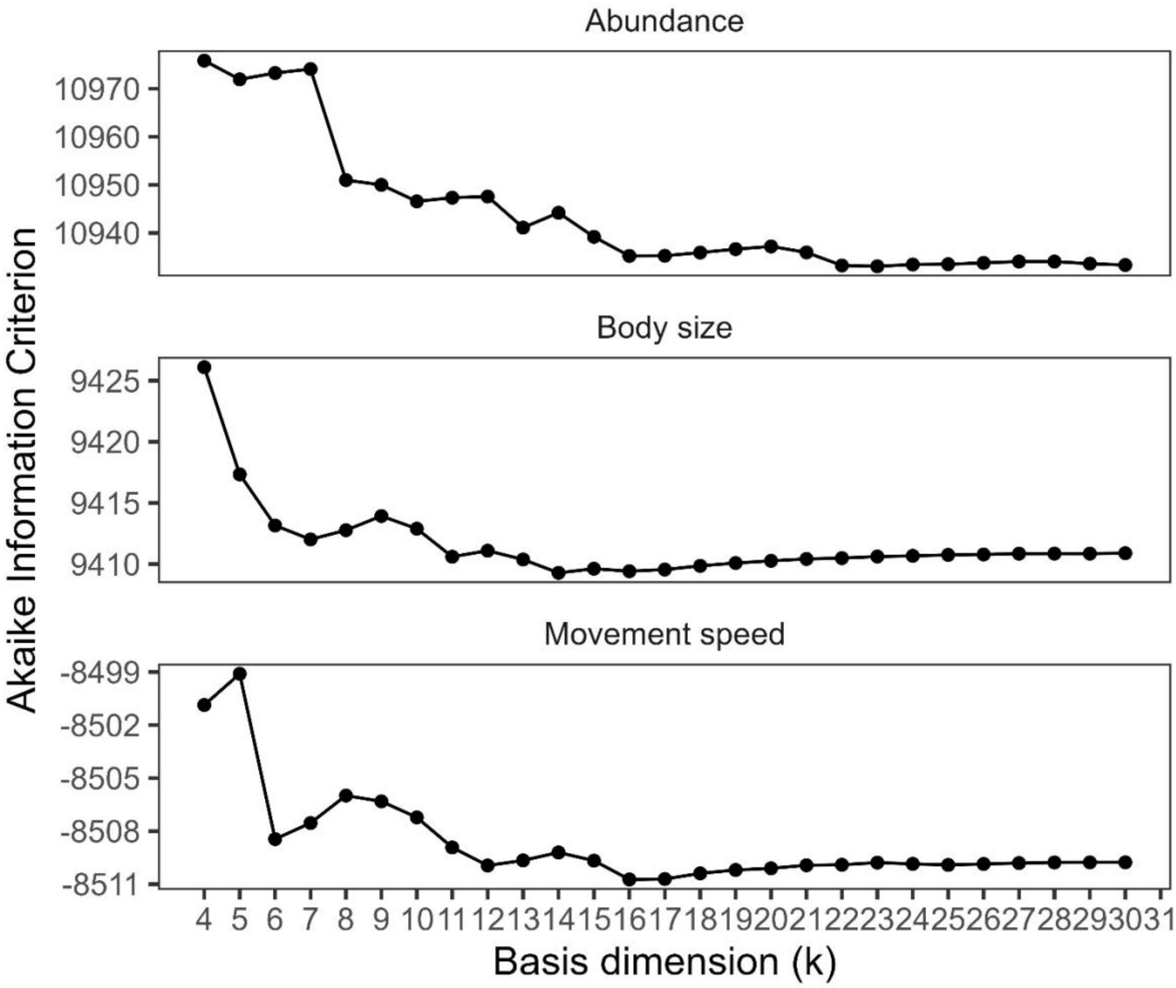

Figure S6. Autocorrelation exploration for timeline components in *P.caudatum*. The solid black line gives a partial autocorrelation of 0, and dashed lines give the significance thresholds for the average time-series length of our observed population replicates. Points are the mean partial autocorrelation coefficients across replicate populations, with associated standard error bars across capturing the variation across populations.

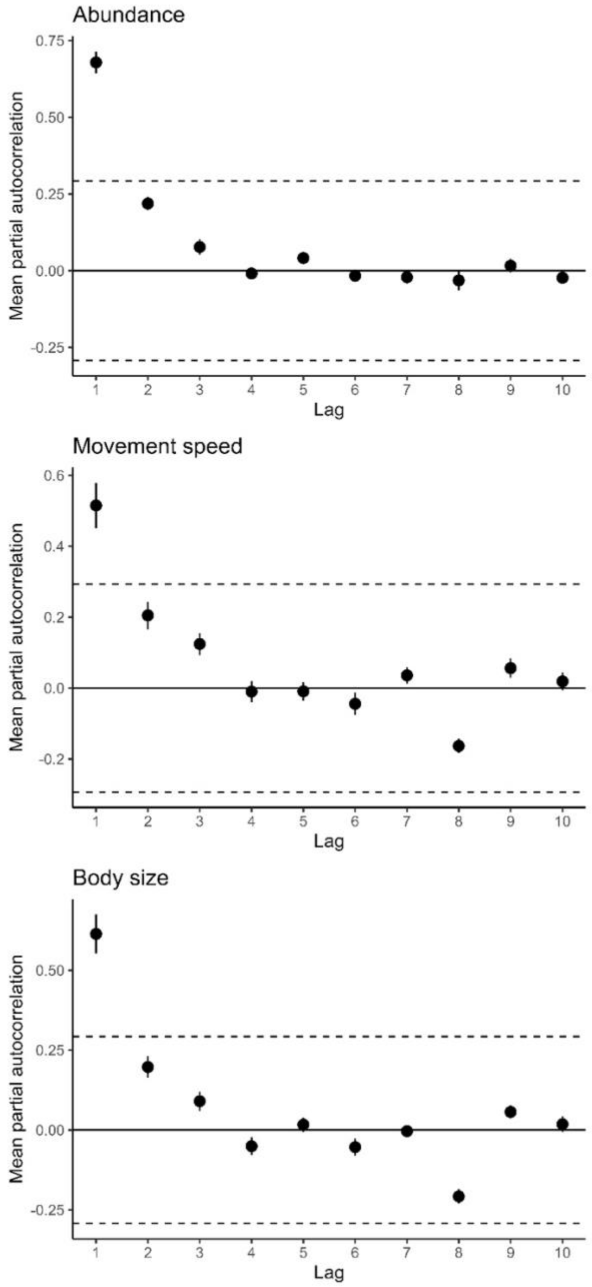

Figure S7. Residual vs. fitted diagnostic plots for each of the nine additive timeseries models for each timeline component in each treatment. Specific model combination (timeline component and treatment) given as the title of each plot. Model structure is given above.

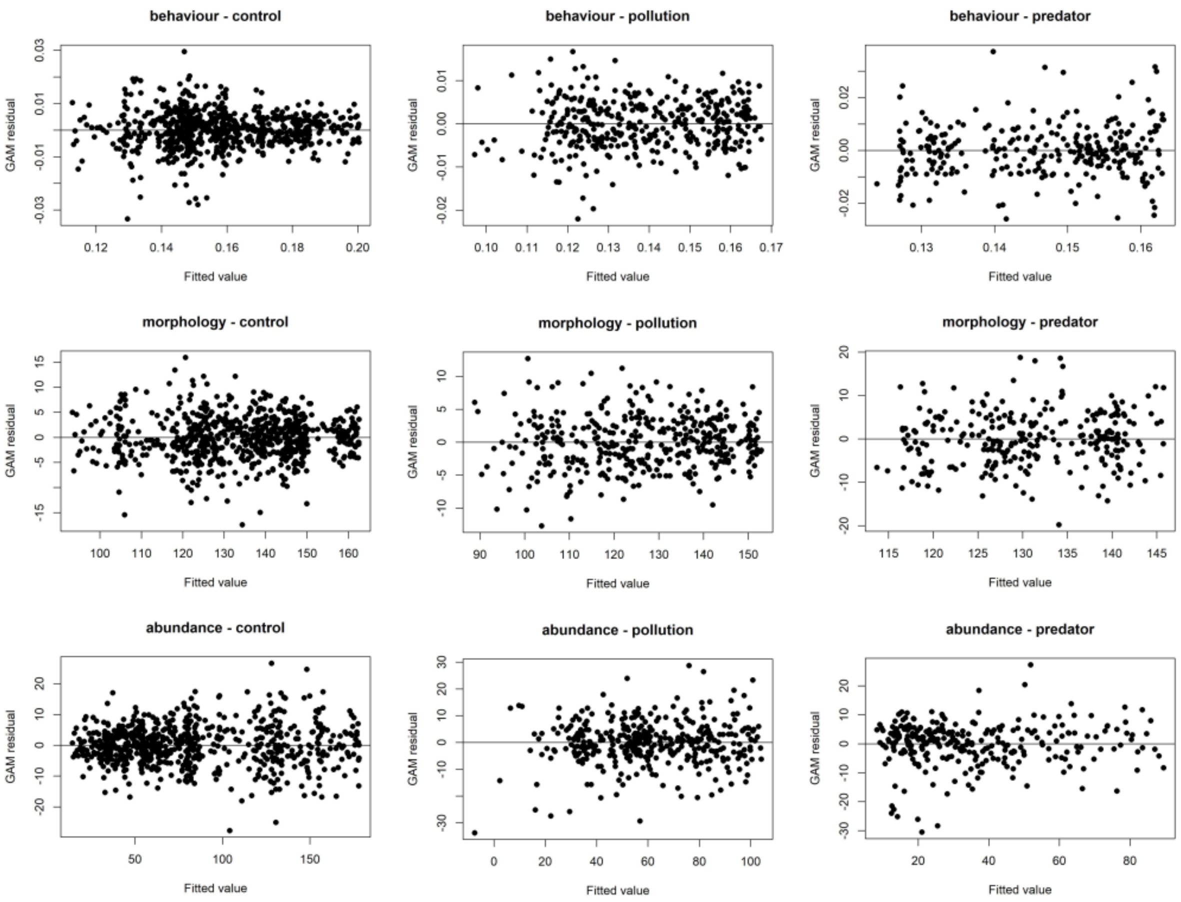

Figure S8. Temporal trends in early warning signal (EWS) metrics of abundance in the Pollution (a) and Predator (b) treatments relative to Control treatments for *Paramecium caudatum.* Columns give three EWS metrics, lag-1 autocorrelation (acf), coefficient of variation (cv), and standard deviation (sd). Dots are point estimates derived from analysis, with GAMM predicted lines and 95% confidence limits. Note that the x axis (the temporal component) is normalised for each replicate using the time before collapse (time before experiment end for control treatments).

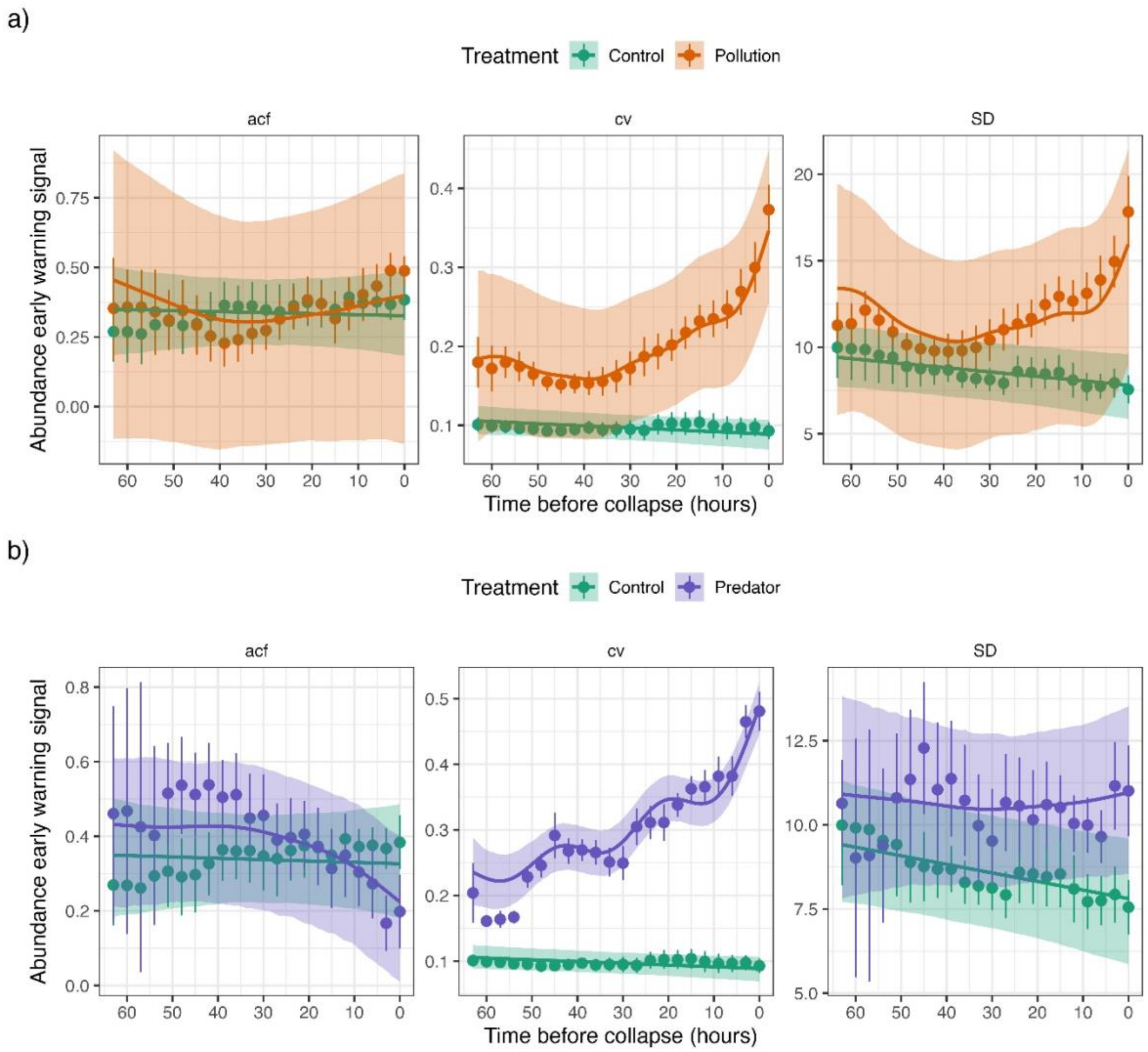

References

McClanahan, T.R., Graham, N.A.J., MacNeil, M.A., Muthiga, N.A., Cinner, J.E., Bruggemann, J.H., *et al.* (2011). Critical thresholds and tangible targets for ecosystem-based management of coral reef fisheries. *Proceedings of the National Academy of Sciences*, 108, 17230–17233.

Pedersen, E.J., Miller, D.L., Simpson, G.L. & Ross, N. (2019). Hierarchical generalized additive models in ecology: an introduction with mgcv. *PeerJ*, 7, e6876.

Pinheiro, J., Bates, D., DebRoy, S., Sarkar, D., Heisterkamp, S., Van Willigen, B., *et al.* (2017). Package 'nlme'. Linear and nonlinear mixed effects models. Available at https://cran.r-project.org/web/packages/nlme/nlme.pdf.

Roth, T., Tresch, S., Du, E. & Braun, S. (2022). Hierarchical change-point regression models including random effects to estimate empirical critical loads for nitrogen using Bayesian Regression Models (brms) and JAGS. *MethodsX*, 9, 101902.

Wood, S.N. (2003). Thin Plate Regression Splines. *Journal of the Royal Statistical Society Series B: Statistical Methodology*, 65, 95–114.

Wood, S.N. (2017). *Generalized Additive Models: An Introduction with R, Second Edition*. CRC Press.
